# Influence of *IL10* (rs1800896) Polymorphism and TNF-α, IL-10, IL-17A, and IL-17F Serum Levels in Ankylosing Spondylitis

**DOI:** 10.3389/fimmu.2021.653611

**Published:** 2021-07-05

**Authors:** Matheus Braga, Fernanda Formaggi Lara-Armi, Janisleya Silva Ferreira Neves, Marco Antônio Rocha-Loures, Mariana de Souza Terron-Monich, Larissa Danielle Bahls-Pinto, Quirino Alves de Lima Neto, Joana Maira Valentini Zacarias, Ana Maria Sell, Jeane Eliete Laguila Visentainer

**Affiliations:** ^1^ Post Graduation Program in Biosciences and Physiopathology, Department of Clinical Analysis and Biomedicine, State University of Maringá, Paraná, Brazil; ^2^ Rheumatology Division, Department of Medicine, State University of Maringá, Paraná, Brazil; ^3^ Immunogenetics Laboratory, Department of Basic and Health Science, State University of Maringá, Paraná, Brazil

**Keywords:** autoimmune disease, spondyloarthritis, rheumatological diseases, interleukin-10, interleukin-17, tumour necrosis factor alpha, single nucleotide polymorphisms, genetic polymorphism

## Abstract

Ankylosing spondylitis (AS) is a chronic autoimmune inflammatory disease that mainly affects the axial and sacroiliac joints. Single-nucleotide polymorphisms (SNPs) in genes encoding cytokines have been associated with AS, which can interfere with the production of these cytokines and contribute to the development of AS. In order to contribute to a better understanding of the pathology of AS, our objective was to investigate a possible association of the *IL10* −1082 A>G SNP (rs1800896) with AS and to evaluate the serum levels of TNF-α, IL-10, IL-17A, and IL-17F in AS patients and controls comparing them with their respective genotypes (*TNF* rs1800629, *IL10* rs1800896, *IL17A* rs2275913, and *IL17F* rs763780). Patients and controls were selected from the Maringá University Hospital and the Maringá Rheumatism Clinic, in Paraná State, Southern Brazil, and they were diagnosed by the ASAS Criteria. In total, 149 patients and 169 controls were genotyped for the *IL10* −1082 A>G polymorphism using a polymerase chain reaction with sequence specific primers (PCR-SSP); the measurement of TNF-α serum levels was performed through the immunofluorimetric test and IL-10, IL-17A, and IL-17F using an ELISA test. There was a high frequency of the *IL10* −1082 G allele in AS patients compared with controls with an odds ratio of 1.83 and 95% confidence interval of 1.32 to 2.54, and a significant difference in the genotype frequencies of the *IL10* −1082 A/G+G/G between patients and healthy controls, with an odds ratio of 3.01 and 95% confidence interval of 1.75 to 5.17. In addition, increased serum levels of IL-10 were observed in AS patients: 2.38 (IQR, 0.91) pg/ml compared with controls 1.72 (IQR 0.93) pg/ml (P = 0.01). Our results also showed an association between *IL17F* rs763780 *C/T+T/T* genotypes and increased serum levels of IL-17F in patients with AS and also in controls. We can conclude that patients with the A/G and G/G genotypes for −1082 A>G (rs1800896) in the *IL10* gene are three times more likely to develop AS, that the serum level of IL-10 was higher in AS patients and that the *IL17F* rs763780 polymorphism can affect the levels of IL-17F in the serum of patients and controls in the same way.

## Introduction

Ankylosing spondylitis (AS) is a chronic autoimmune inflammatory disease that mainly affects the axial and sacroiliac joints, causing severe pain. In more advanced cases, this inflammation can lead to fibrosis and calcification, spinal fusion, and consequently, a loss of flexibility (1, 2). Although the major histocompatibility complex (MHC) class I allele HLA-B*27 is strongly associated with AS, genome-wide association studies have revealed a number of other risk factors for the disease, including several innate immune-related pathways and cytokines (3).

IL-10 is an immunomodulatory cytokine encoded by the *IL10* gene on chromosome 1q31–32, containing five exons separated by four introns (4). Polymorphisms located in the 5′-flanking region of the *IL10* gene, at positions −1082 A>G, −819 T>C, and −592 A>C, are known to be involved in regulating the production of IL-10, with the first being the best characterised of them (5).

Three single nucleotide polymorphisms (SNPs) in genes that encode inflammatory cytokines have been associated with AS, in a previous study by our research group (*TNF* rs1800629, *IL17A* rs2275913, and *IL17F* rs763780) (6). These polymorphisms can interfere with the level of production of these cytokines and contribute to the development of AS (7–10).

Inflammatory cytokines, such as tumour necrosis factor alpha (TNF-α) and interleukin 17 (IL-17), are known to be involved in AS (11, 12). TNF-α is a key component of the immune system and a potent pro-inflammatory cytokine that is highly produced after infection or tissue damage (13) and is present in high concentrations in patients with AS (12). IL-17 is a pro-inflammatory cytokine that contributes to the pathogenesis of several inflammatory diseases. The IL-17 family consists of six structurally related cytokines (IL-17A, IL-17B, IL-17C, IL-17D, IL-17E, and IL-17F) (14). In rheumatological diseases, this interleukin causes cartilage damage in an experimental animal model (15).

The pathogenesis of AS has not been fully elucidated, and some cytokines may play a key role in the disease. In order to elucidate the pathology of AS and identify new genetic markers, we investigated a possible association of the *IL10* SNP −*1082 A>G* (rs1800896) with AS, and evaluated the serum levels of TNF-α, IL-10, IL-17A, and IL-17F in AS patients and controls compared with their respective genotypes (*TNF* rs1800629, *IL17A* rs2275913, *IL17F* rs763780, and *IL10* rs1800896).

## Methodology

### Study Population

Unrelated patients were diagnosed by the ASAS Criteria 2009 (16) for axial spondyloarthritis (SpA) and the ASAS Criteria 2011 (17) for peripheral SpA. Patients were selected from the Maringá University Hospital-Maringá State University and the Maringá Rheumatism Clinic, in Paraná State, Southern Brazil. All patients had magnetic resonance imaging of the sacroiliac joints and were evaluated for the presence of HLA-B*27. In addition, individuals were selected to form the control group, following the criteria for inclusion: no autoimmune and/or rheumatic diseases, unrelated to the patient group, and belonging to the same ethnic group as the patients, and from the same region. This research was approved by the Standing Committee of Ethics of the State University of Maringá (CAAE 687.222/2014), and all participants signed an informed consent form.

### Sample Collection and DNA Extraction

Peripheral blood was collected from case and control groups using tubes without anticoagulants for the determination of serum levels of cytokines and using EDTA-anticoagulant for DNA extraction. Genomic DNA was extracted from blood samples using the extraction kit Biopur^®^ (BIOMETRIX Diagnostic, Curitiba, Paraná, Brazil), according to the manufacturer’s recommendations.

### Determination of *IL10* Gene Polymorphism

One hundred and forty-nine patients and 169 controls were genotyped for the *IL10* −1082 A>G polymorphism using a polymerase chain reaction with sequence specific primers (PCR-SSP), and a methodology adapted from (18). The primer sequences used are shown in **Table 1**.

**Table 1:** Primer sequences.

Primers	Sequence	Bp	Ref
*IL10*		550 bp	(18)
Common	5′-CAGCCCTTCCATTTTACTTTC-3′		
G allele	5′-TACTAAGGCTTCTTTGGGAG-3′		
A allele	5′-CTACTAAGGCTTCTTTGGGAA-3′		
*HGH*		431 bp	(19)
*HGH* F	5′-TGCCTTCCCAACCATTCCCTTA-3′		
*HGH* R	5′-CAACTCACGGATTTCTGTTGTGTTTC-3′		

Bp, base pairs; Ref, reference; HGH, human growth hormone.

The protocol was adapted to a final volume of 10 µL of the reaction mixture containing 50 ng of DNA template, 1.8 ng/μM of each *IL10* primer, 0.75 ng/µM of each Human growth hormone (HGH) for internal control, 0.19 mM of dNTP mixture, 1.5 mM of MgCl_2_ for allele G reaction and 2.0 mM for allele A reaction, 0.47× Reaction Buffer, and 0.55 U of Taq DNA polymerase.

PCR conditions for amplification included an initial melting step of 5 min at 94°C, followed by 32 cycles of 40 s at 95°C, 50 s at 65°C, 40 s at 72°C, and a final elongation at 72°C for 7 min. After amplification, PCR products were visualised in a 2% agarose gel stained with SYBR™ Safe DNA Gel Stain dye (Invitrogen, Carlsbad, CA, USA) using the Quantum ultraviolet gel documentation system.

**Figure 1:**
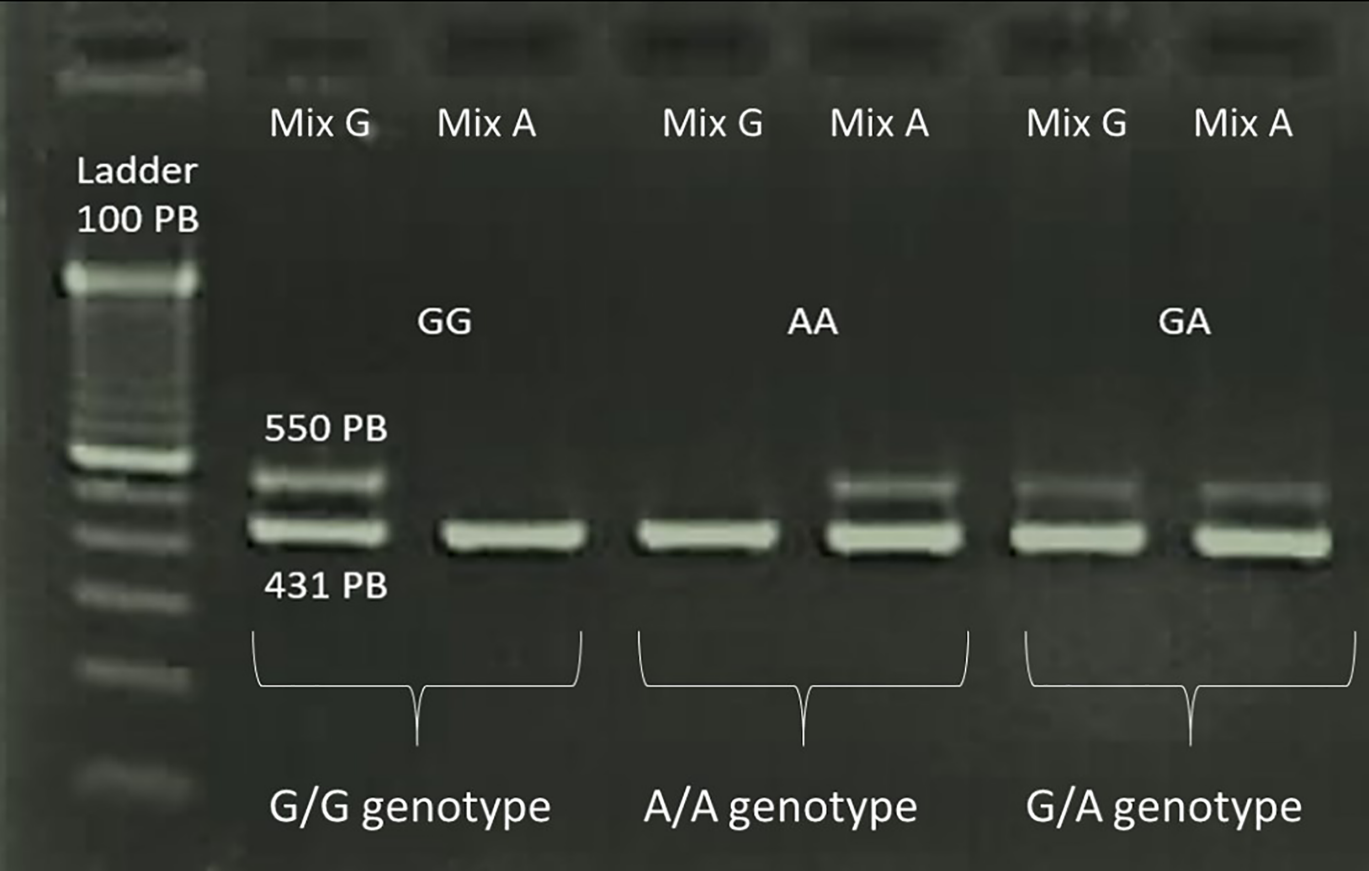
Amplified products of the PCR-SSP assay for analysing the −1082 A>G polymorphism of *IL10* visualised in 2% agarose gel. Bands of 550 bp correspond to the *IL10* fragment and bands of 431 bp correspond to the *HGH* fragment, which was used as an internal control for the reaction.

For methodology validation, samples previously genotyped in our laboratory using a commercial kit (One Lambda Cytokine Genotyping Primer Pack, One Lambda) were used as positive controls for each of the three genotypes obtained (A/A, G/A, and G/G) (**Figure 1**).

### Determination of Serum Levels of Cytokines

The samples selected for cytokine measurement were randomly chosen, paired by age, gender, and genotype. For IL-10, 28 AS patients and 20 controls genotyped for *IL10* rs1800896 were selected, with 12 being A/A, and 16 being A/G+G/G for AS and 10 being A/A, and 10 being A/G+A/A for controls. *TNF, IL17A*, and *IL17F* gene polymorphisms were previously determined by our group for the same samples and the polymorphisms analysed were *TNF* rs1800629, *IL17A* rs2275913, and *IL17F* rs763780 (6). 35 patients were selected for the analysis of TNF-α levels, with 17 being G/G, and 20 being G/A+A/A for AS, and 15 controls were also included, of which 8 were G/G and 7 were G/A+A/A. For the IL-17A analysis, 45 AS patients, including 21 G/G and 24 G/A+A/A, and 15 controls, including 5 G/G and 10 G/A+A/A, were selected. For the IL-17F analysis, 40 patients were selected, with 24 being T/T, and 16 being C/T+C/C; for the 14 controls, there were seven T/T and seven C/T+C/C. The characteristics of AS patients and controls selected for measurement of cytokines are shown in **Table 2**. There were no significant differences between the patients with AS and controls for gender and age distribution for any group of cytokines (P > 0.05).

**Table 2:** Characteristics of AS patients and controls selected for cytokines measurement.

Cytokine	Age		P value
	Patients	Controls	
TNF-α	44.32^a^ ± 14.39	43.33^a^ ± 11.02	0.33^b^
IL-10	46.03^a^ ± 14.70	44.45^a^ ± 11.74	0.30^b^
IL-17A	45^a^ ± 14.96	42.87^a^ ± 13.60	0.79^b^
IL-17F	43.73^a^ ± 15.44	41.64^a^ ± 9.20	0.05^b^
	**Gender (M/F)**		
	**Patients**	**Controls**	
TNF-α	22/15	8/7	0.68^c^
IL-10	19/15	15/5	0.99^c^
IL-17A	24/21	6/9	0.37^c^
IL-17F	21/19	8/6	0.76^c^

a Average age; ± standard deviation.

b Statistical analysis was performed by two-sample t test.

c Statistical analysis was performed by OpenEpi two-by-two tables χ^2^ test.

The determination of serum levels of TNF-α was performed using the Kit Luminex Technology with the ProcartaPlex™ Immunoassay (Invitrogen, ThermoFisher Scientific, Inc., Burlington, Ontario, Canada). The determination of IL-10 serum levels was performed using the human IL-10 uncoated ELISA kit (Affymetrix eBioscience, North America, USA). The determination of serum levels of IL-17A was performed using the Human IL-17A platinum ELISA Kit (Affymetrix eBioscience, North America, USA), while the determination of serum levels of IL-17F cytokine was performed using the Human IL-17F ELISA Kit (Affymetrix eBioscience, North America, USA). The minimum detection limit for each kit was 0.5 picograms per ml (pg/ml) for TNF-α, 1.6 pg/ml for IL-10, 0.5 pg/ml for IL-17A, and 3.3 pg/ml for IL-17F.

### Statistical Analysis

The QUANTO 1.2.4 software (http://biostats.usc.edu/software) was used to calculate the sample size using the following values: prevalence of the less frequent allele 0.30%, population risk 0.01%, power 0.80, and OR >3.0. For genetic statistical analyses and to estimate the distribution of genotype frequencies according to the Hardy-Weinberg equilibrium (HWE), we applied the software SNPStats (available at: https://www.snpstats.net/start.htm) (20), which was also used for detecting the odds ratio balance, with a 95% confidence interval (CI) after adjustment for age and HLA*B27 marker. The Akaike information criterion (AIC) was used to choose the inheritance model that best fits the data. The OpenEpi program was used to calculate the allele association using the two-by-two tables test (https://www.openepi.com/Menu/OE_Menu.htm) (21).

To compare the serum levels of cytokines with their respective genotypes, the BioEstat 5.0 program was used. The Shapiro-Wilk test was used to calculate the normality of the samples and the Mann-Whitney test to compare the samples. All values of serum concentration cytokines were expressed as median and Interquartile range (IQR); differences were considered statistically significant if P<0.05.

## Results

The characteristics and clinical data of individuals genotyped for the *IL10* −1082 A>G polymorphism are shown in **Table 3**. There were no significant differences between AS patients and controls for gender distribution (P>0.05). HLA-B*27 was previously analysed using PCR-SSP according to the method published by our research group (22) and it was more frequent in patients with AS than in controls (P<0.0001).

**Table 3:** Characteristics of AS patients and healthy controls.

Characteristics	AS = 149	Controls = 169	P value
**Gender (M/F)**	72/77	69/100	0.17^b^
**Age (years)**	46.6 ± 15.4^a^	40.6 ± 12.1^a^	0.002^c^
HLA-B*27	64 (43%)	11 (6%)	<0.0001^b^
**BASDAI (>4/<4)**	110/39		
**Sacroiliitis**	133 (89%)		
**Good response to NSAID**	12 (8%)		
**Family history of SpA**	29 (19%)		
**Elevated CRP**	5 (3%)		
**Arthritis**	27 (18%)		
**Enthesitis**	56 (38%)		
**Uveitis**	5 (3%)		
**Inflammatory bowel disease**	6 (4%)		

a Mean ± standard deviation (all such values).

b Statistical analysis was performed by OpenEpi Two by Two tables χ^2^- test.

c Statistical analysis was performed by two-sample t test.

AS, ankylosing spondylitis; BASDAI, bath ankylosing spondylitis disease activity index; NSAID, non-steroidal anti-inflammatory drugs; SpA, spondyloarthritis; CPR, c-reactive protein.

Determination of the *IL10* gene polymorphism by PCR-SSP demonstrated an increased frequency of the −1082 G allele in AS patients compared to controls (**Table 4**). In addition, there was significant difference in the genotype frequency of the *IL10* −1082 A>G polymorphism between patients and controls. To estimate the association between the genotype and AS occurrence, the disease odds ratio (OR) and 95% confidence interval (CI) were assessed and adjusted for age and HLA*B27 marker. The results suggest that the −1082 A/G and G/G genotypes were associated with disease in a dominant model with an increased risk for AS (OR, 3.01; 95% CI, 1.75–5.17). The *IL10* genotype frequency distributions were in Hardy-Weinberg equilibrium in patients and controls (P>0.05).

**Table 4:** Allele and genotype frequency distribution for *IL10* −*1082 A>G* and the association with the risk for ankylosing spondylitis, regardless of HLA-B*27 marker and age in different genetic models.

*IL10* −1082	AS	Controls	OR (95% CI)	P value	AIC
	N = 149	N = 169			
**Codominant**	**n (f)**	**n (f)**		0.0002	358.1
A/A	40 (0.27)	85 (0.5)			
A/G	83 (0.56)	63 (0.37)	3.05 (1.72–5.41)^a^		
G/G	26 (0.17)	21 (0.12)	2.87 (1.31–6.29)^a^		
**Dominant**	**n (f)**	**n (f)**		<0.0001	356.1*
A/A	40 (0.27)	85 (0.5)			
A/G – G/G	109 (0.73)	84 (0.50)	3.01 (1.75–5.17)^a^		
**Recessive**	**n (f)**	**n (f)**		0.23	371.5
A/A- A/G	124 (0.83)	147 (0.87)			
G/G	25 (0.17)	22 (0.13)			
**Alleles**	**n (f)**	**n (f)**		0.0002	
A	163 (0.55)	233 (0.69)			
G	135 (0.45)	105 (0.31)	1.83 (1.32–2.54)^b^		

*Best inheritance model defined according to 20.

a Statistical analysis was performed by SNPStats software.

b Statistical analysis was performed by OpenEpi Two by Two tables. f: genotype or allele frequency.

AS, ankylosing spondylitis; OR, odds ratio; CI, confidence interval; AIC, criterion Akaike information.

Results of the analysis of the association between *IL10* −1082 A>G (rs1800896) polymorphism with different clinical data of AS are shown in **Table 5**. There was no statistical difference between *IL10* genotypes or alleles in relation to clinical data of AS. The smaller number of patients in each subgroup could be related to these results.

**Table 5:** Analysis of *IL10* gene polymorphisms (rs1800896) in AS patients separated according to clinical data.

Clinical data		Genotypes (n)				Alleles		
		AA	AG	GG	P1	A	G	P2
BASDAI (>4/<4)								
	> 4	28	62	20	0.70	118	102	0.53
	< 4	12	21	6		45	33	
Sacroiliitis								
	Positive	35	72	26	0.14	142	124	0.18
	Negative	5	11	0		21	11	
Good response to NSAID								
	Positive	1	8	3	0.30	10	14	0.18
	Negative	39	75	23		153	121	
Elevated CRP								
	Positive	1	3	1	0.93	5	5	0.76
	Negative	39	80	25		158	130	
Arthritis								
	Positive	7	15	5	0.98	29	25	0.87
	Negative	33	68	21		134	110	
Enthesitis								
	Positive	15	33	8	0.60	63	49	0.68
	Negative	25	49	18		99	85	
Uveitis								
	Positive	0	4	1	0.37	4	6	0.34
	Negative	40	79	25		159	129	
Inflammatory bowel								
	Positive	3	3	0	0.30	9	3	0.14
	Negative	37	80	26		154	132	

P1: Patients positive versus patients negative using 3×2 contingency table.

P2: Patients positive versus patients negative using 2×2 contingency table.

AS, ankylosing spondylitis; BASDAI, bath ankylosing spondylitis disease activity index; NSAID, non-steroidal anti-inflammatory drugs; SpA, spondyloarthritis; CPR, c-reactive protein.

**Figure 2:**
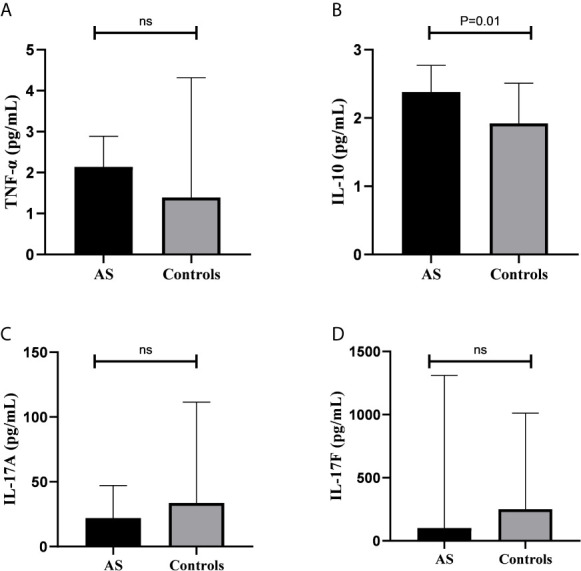
Comparison between patients and controls for cytokine levels. **(A)** Comparison of TNF-α levels between patients and controls; **(B)** Comparison of IL-10 levels between patients and controls; **(C)** Comparison of IL-17A levels between patients and controls; **(D)** Comparison of IL-17F levels between patients and controls. AS, ankylosing spondylitis; ns, not significant.

The analyses of serum cytokine levels in AS patients and controls are shown in **Figure 2**. Higher serum levels of IL-10 were observed in patients, as the levels of IL-10 was 2.38 (IQR 0.91) pg/mL in patients and 1.72 (IQR, 0.93) pg/ml in controls (P=0.01) (**Figure 2B**). There were no statistically significant differences in the serum levels of the other cytokines when patients were compared to controls. The serum levels of the cytokines in patients were as follows: TNF-α 2.14 (IQR, 3.65) pg/ml, IL-17A 22.12 (IQR, 51.97) pg/ml, and IL-17F 100.13 (IQR, 1273.65) pg/ml. For controls, the serum levels were as follows: TNF-α 1.39 (IQR, 3.14) pg/ml; IL-17A 33.50 (IQR, 339) pg/ml, and IL-17F 251.04 (IQR, 765.45) pg/ml.

**Figure 3:**
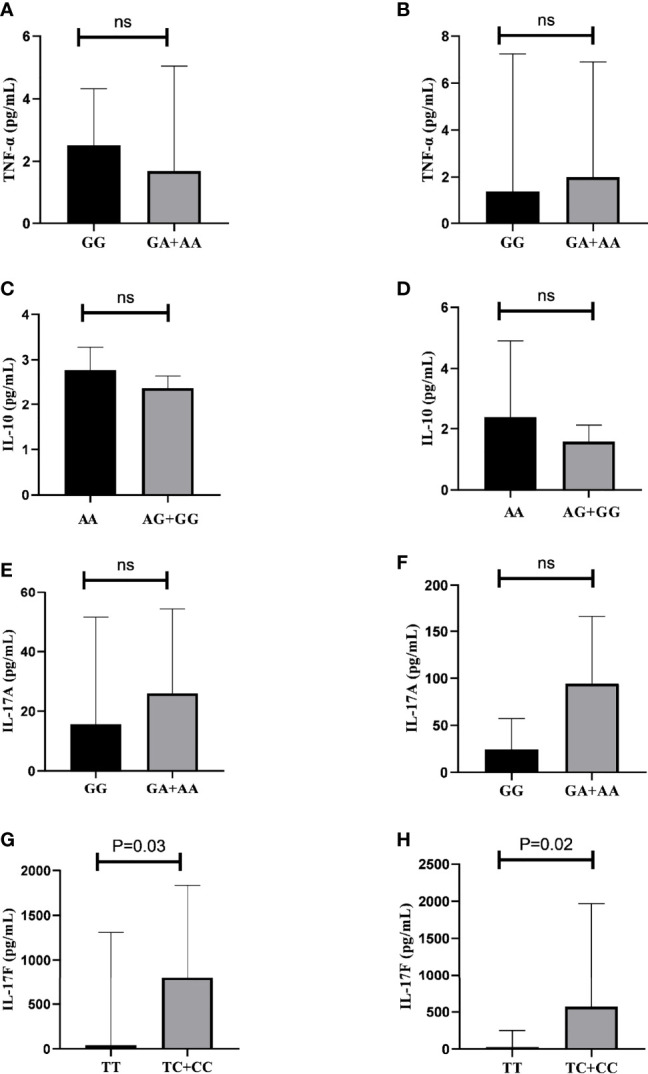
Relation between serum cytokine levels and genotypes in AS patients and controls. **(A)** Comparison of TNF-α levels in patients with *TNF* rs1800629 G/G vs. G/A+AA genotypes. **(B)** Comparison of TNF-α levels in controls with *TNF* rs1800629 G/G *vs.* G/A+A/A genotypes. **(C)** Comparison of IL-10 levels in patients with *IL10* rs1800896 A/A *vs.* A/G+G/G genotypes. **(D)** Comparison of IL-10 levels in controls with *IL10* rs1800896 A/A *vs.* A/G+G/G genotypes. **(E)** Comparison of IL-17A levels in patients with *IL17A* rs2275913 G/A+A/A *vs.* G/G genotypes. **(F)** Comparison of IL-17A levels in controls with *IL17A* rs2275913 G/A+A/A *vs.* G/G genotypes. **(G)** Comparison of IL-17F levels in patients with *IL17F* rs763780 T/C+C/C *vs.* T/T genotypes. **(H)** Comparison of IL-17F levels in controls with *IL17F* rs763780 T/C+C/C *vs.* T/T genotypes; ns, not significant.

The analyses of TNF-α, IL-10, IL-17A, and IL-17F serum cytokine levels with genotypes in AS patients and controls are shown in **Figure 3**. Higher serum levels of IL-17F were observed in patients with the *IL17F* rs763780 T/C+C/C genotypes [796.04 (IQR, 1496.23) pg/ml compared to patients with the T/T genotype 43.77 (IQR, 384.1) pg/ml] (p=0.03) (**Figure 3H**). The same association was found for controls: T/C+C/C 570.10 (IQR, 1325.23) pg/ml compared with *T/T* 25.36 (IQR, 142.73) pg/ml (P=0.02) (**Figure 3I**).

No statistically significant difference was found for the other cytokines. TNF-α serum level in AS patients carrying *TNF* rs1800629 G/A+A/A was 1.70 pg/ml (IQR, 4.71) and 2.51 (IQR, 2.21) pg/ml for those carrying G/G (**Figure 3A**). For controls, it was 2.00 (IQR, 4.73) pg/ml for those carrying G/A+A/A and 1.39 (IQR, 1.37) pg/ml for G/G (**Figure 3B**). Serum levels of IL-10 in AS patients carrying *IL10* rs1800896 A/G +A/A were 2.38 (IQR, 0.45) pg/ml and 2.77 (IQR 0.91) pg/mL in those carrying A/A (**Figure 3C**); for controls carrying A/G +A/A, it was 1.58 (IQR, 0.68) pg/ml and for A/A 2.12 (IQR 1.19) pg/ml (**Figure 3D**). Serum levels of IL-17A were 26.00 (IQR, 51.6) pg/ml in AS patients carrying the *IL17A* rs2275913 G/A+A/A genotypes and 15.63 (IQR, 49.6) pg/ml for those carrying the G/G genotype (**Figure 3E**); for controls, it was 94.63 (IQR, 129.23) pg/ml for individuals carrying G/A+A/A and 24.37 (IQR, 30.43) pg/ml for those carrying G/G (**Figure 3F**).

## Discussion

In this study, we found that the *IL10* −1082 G allele and the A/G+G/G genotypes (in a dominant inheritance model) were associated with the risk of AS. It is worth mentioning that our analyses of the IL10 -1082 polymorphism were adjusted for age and presence of HLA-B*27, and that we found this association to be independent of these factors, which makes the evidence of an association between the IL10 gene and AS more powerful.

Despite the population difference, another study conducted with a Chinese population also found an association with the same polymorphism and AS (23). The Paraná population mainly consists of people of European origin (80.6%), with a small contribution from African (12.5%), and Indigenous (7.0%) populations (24, 25). Hence, patients and healthy controls in our study were classified as mixed ethnic groups, according to phenotypic characteristics.

Our study also showed higher serum levels of IL-10 in AS patients compared to controls, similarly to the Chinese study (23). Increased levels of IL-10 and IL-10–positive T cells were detected in synovial fluid from AS patients in other studies (26, 27). Although these results seem controversial because of IL-10 being an anti-inflammatory cytokine, a way to better understand the role of IL-10 in the pathogenesis of disease can be through the M2 macrophages. Some studies have shown an increase in M2 CD163+ in synovial biopsies and inflamed colon of the patients with AS, and CD163 synovial over-expression has been shown to be correlated with disease (28, 29); in this context, IL-10 can alter the phenotype of macrophages from M1 to M2 (30). Furthermore, it has been proposed that the M2 macrophages express less IFN-γ, leading to an increased Th17 response since IFN-γ can suppress IL-17 expression (31), and it has been reported that the IL-17 is one of the main cytokines involved in the pathogenesis of AS (28, 29).

In this study, we found no statistical differences between the levels of TNF-α, IL-17A, and IL-17F among patients and controls, despite some studies having shown an increase in inflammatory cytokines, such as IL-17 and TNF-α in AS (32, 33). We must take into account that our patients were being treated with non-steroidal anti-inflammatory drugs (NSAIDs), and anti-TNF therapy for longer than 6 months.

In order to better understand the influence of SNPs associated with AS, *IL10* rs1800896 in the present study, as well as *TNF* rs1800629, *IL17A* rs2275913, *IL17F* rs763780 that were associated with the risk of developing AS in a previous study by our group (6), we compared the serum levels of the cytokines between the risk genotypes and non-risk genotypes in patients and controls.

With regard to TNF-α, we found no differences in the serum levels of this cytokine between the *TNF* rs1800629 genotype G/G compared to the risk genotypes G/A+A/A. The G/A+A/A genotypes have also been reported to be major producers of TNF-α in congenital heart disease (34). The A allele was found to be the higher producer of TNF-α in an *in vitro* study (35). Although we did not observe this association, we should also consider the use of anti-TNF in the treatment.

Many studies show that the *IL10* −*1082* genotypes A/G+G/G were the largest producers of IL-10 (23, 36–38). Despite the association of A/G+G/G genotypes with AS and increased levels of IL-10 in patients, we did not find any association between these genotypes and the levels of IL-10, suggesting that other SNPs could influence the production of the cytokine.

We also found no differences in the serum levels of IL-17A cytokine between G/G and G/A+A/A genotypes. The genotypes G/A and A/A have been reported to be associated with the increased production of IL-17A in a recent study on coronary disease (39). However, this relationship is controversial, as other studies have shown an increase in IL-17A in patients with the A/A genotype (40), a lack of association with this SNP and production (41), and high levels in individuals carrying the genotypes GG+GA (42). These differences could suggest that the polymorphism (rs2275913) does not influence IL-17A levels.

The assessment of the association between IL-17F levels and the *IL17F* T>C (rs763780) polymorphism showed an association between increased IL-17F production and the T/C+C/C genotypes in patients and controls. These results suggest that variations in the sequence of the specific nucleotides of *IL17F* can have a direct effect on the production of the encoded protein, IL-17F. Another study with psoriasis also reported a higher level of IL-17F in patients with the T/C+C/C genotypes (43).

However, we must take into account some limitations of our study. It is a cross-sectional study, although the association between the polymorphism (rs763780) and IL-17F levels has been confirmed. It was not possible to establish a causal relationship, requiring experimental studies and analysis of cytokines in tissue and synovial fluid samples. Another weakness of our study was the impossibility of using patients who had not yet used any type of medication.

We can conclude that the *IL10* −1082 G allele and the A/G+G/G genotypes (in a dominant inheritance model) were risk factors for AS, regardless of HLA-B*27 and age, and that serum IL-10 levels were increased in patients with AS compared to controls.

## Data Availability Statement

The original contributions presented in the study are included in the article/supplementary material. Further inquiries can be directed to the corresponding author.

## Ethics Statement

This research was approved by the standing committee of ethics of the State University of Maringá (CAAE 687.222/2014). The patients/participants provided their written informed consent to participate in this study.

## Author Contributions

MB executed the research protocol, analyzed the data, and wrote the manuscript. MT-M, LB-P and JZ contributed to the writing and critical analysis of the manuscript. JN and MR-L contributed to the collection of data, sample and classification of patients. FL-A contributed to the implementation of the protocols. QN supervised the analyses and the execution of this research. AS and JV developed the original idea and provided intellectual input and critical analysis of the manuscript. All authors contributed to the article and approved the submitted version.

## Funding

This work was supported by the Laboratory of Immunogenetics (Proc. n.1589/2017-CSD-UEM).

## Conflict of Interest

The authors declare that the research was conducted in the absence of any commercial or financial relationships that could be construed as a potential conflict of interest.
